# Calcium/strontium chloride impregnated zeolite A and X granules as optimized ammonia sorbents†

**DOI:** 10.1039/d2ra02981b

**Published:** 2022-12-07

**Authors:** Zhejian Cao, Xiaoping Cai, Ana Carolina Feltrin, Peizhong Feng, Andreas Kaiser, Farid Akhtar

**Affiliations:** Division of Materials Science, Luleå University of Technology 971 87 Luleå Sweden zhejian@chalmers.se farid.akhtar@ltu.se; School of Materials Science and Physics, China University of Mining and Technology 221116 Xuzhou People's Republic of China; Department of Energy Conversion, Technical University of Denmark 2800 Kgs. Lyngby Denmark

## Abstract

Calcium chloride (CaCl_2_) impregnated zeolite A and strontium chloride (SrCl_2_) impregnated zeolite A and X composite granules were evaluated as ammonia sorbents for automotive selective catalytic reduction systems. The SrCl_2_-impregnated zeolite A granules showed a 14% increase in ammonia uptake capacity (8.39 mmol g^−1^) compared to zeolite A granules (7.38 mmol g^−1^). Furthermore, composite granules showed 243% faster kinetics of ammonia sorption (0.24 mmol g^−1^ min^−1^) compared to SrCl_2_ (0.07 mmol g^−1^ min^−1^) in the first 20 min. The composite CaCl_2_/SrCl_2_ impregnated zeolite A granules combined the advantages of the zeolites and CaCl_2_/SrCl_2_, where the rapid physisorption from zeolites can reduce the ammonia loading and release time, and chemisorption from the CaCl_2_/SrCl_2_ offers abundant ammonia capacity. Moreover, by optimizing the content of SrCl_2_ loading, the composite granules maintained the granular form with a crushing load of 17 N per granule after ammonia sorption–desorption cycles. Such structurally stable composite sorbents offer an opportunity for fast ammonia loading/release in automotive selective catalytic reduction systems.

## Abstract 2

Zeolite–alkaline earth metal halide composites demonstrate a combination of physisorption and chemisorption as an ammonia sorbent, with a stable structure in the form of granules.

## Graphical abstract

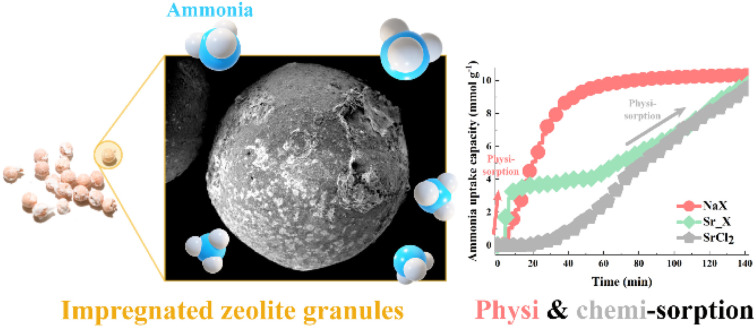

## Introduction

Air pollution has been a chronic problem and raised increasing concerns since the coronavirus disease 2019 (COVID-19) pandemic.^1–3^ Nitrogen dioxide (NO_2_), as one of the six critical air pollutants according to the United States Environmental Protection Agency (EPA), can result in severe respiratory diseases and devastating environmental issues, such as acid rain, smog, fine particulate matter (PM_2.5_), *etc.*^4,5^ Therefore, nitrogen oxide reduction (deNO_*x*_) has been a long-term goal with increasingly strict emission standards worldwide. Selective catalytic reduction (SCR) is one of the most common approaches to eliminate NO_*x*_. In SCR systems, NO_*x*_ is reduced by ammonia (NH_3_) to environment-friendly water (H_2_O) and nitrogen (N_2_).^6^ However, NH_3_ as a hazardous gas has faced several challenges in its storage and release in automotive SCR systems.^7,8^

Conventional urea-based SCR systems utilize urea ((NH_2_)_2_CO) as an indirect ammonia source, requiring high exhaust temperature and producing carbon dioxide (CO_2_) as a byproduct during the hydrolysis reaction.^9^ Furthermore, with a series of problems, such as freezing at low temperatures and catalyst poisoning by the urea residuals, urea-based SCR systems have been replaced by solid SCR systems in several countries.^10–12^ In solid SCR systems, NH_3_ is stored in solid form, typically in the form of metal ammine complexes by alkaline earth metal halides (AEMHs).^13,14^ The AEMHs demonstrate excellent ammonia storage capacity.^15^ With direct ammonia dosing, the deNO_*x*_ efficiency of the solid SCR system has been enhanced at low exhaust temperatures.^14^ Therefore, various research and applications on AEMHs as ammonia carriers have been studied, including hydrogen storage, heat pumps, *etc.*^16–19^ Nevertheless, AEMHs as ammonia carriers emerge several shortcomings impeding the applicable scope. For instance, CaCl_2_ and SrCl_2_ expand up to 4 times by volume after complete ammonia absorption and generate 70% porosity.^20,21^ These dramatic volume changes can result in the disintegration of the structured AEMHs into powder. In automotive SCR systems, this poor structural stability of the ammonia carriers can lead to safety risks in the vehicles and uncontrollable ammonia dosing performance, such as inefficient use of space and pressure drop.^22,23^ Moreover, it has been reported that in these applications AEMHs with slow kinetics require considerable time (up to 36 hours for CaCl_2_) to achieve a complete ammonia sorption cycle.^24^ Slow kinetics of ammonia absorption and desorption in AEMHs can result in long ammonia loading and release time; especially in the first 5–10 min when the vehicles have a cold start where the tank of ammonia sorbents is far below the threshold temperature, the long ammonia release time can lead to unexpected NO_*x*_ escape, which can hinder the development of the NO_*x*_ emission standard.^23,25,26^ Materials providing rapid ammonia sorption and releasing kinetics, therefore, is of desire to elevate the performance of SCR systems.

Microporous materials, including zeolites, metal–organic frameworks (MOFs), activated carbon, *etc.*, have been widely studied for gas adsorption applications owing to their high specific surface area.^27–30^ Among them, zeolites A (Linde type A) and X (faujasite type X) have been intensively investigated due to their excellent chemical/thermal stability, and industrial maturity. By tailoring the zeolites pore opening with the ion-exchange method, the gas uptake capacity and separation can be modified for specific applications, such as methane (CH_4_)/CO_2_ separation in biogas.^31,32^ However, the study of zeolites as ammonia carriers have been rarely discussed due to their relatively low ammonia uptake capacities (9.3 mmol g^−1^ in NaX, 7.8 mmol g^−1^ in CaA) compared to AEMHs (63.0 mmol g^−1^ in CaCl_2_, 50.5 mmol g^−1^ in SrCl_2_).^15,33–35^ Ammonia sorption mechanisms are reported to be different in zeolites compared to AEMHs. In AEMHs, ammonia molecules are strongly absorbed *via* the formation of coordination complexes in a chemisorption process. For example, CaCl_2_ absorbs 8 ammonia molecules according to eqn (1)–(3), where the ammonia desorption energy (69 kJ mol^−1^) is higher than 40 kJ mol^−1^.^15,36–38^ Zeolites, as physisorbents, absorb ammonia with weak interaction, where the major part of the ammonia molecules are released with desorption energies below less than 40 kJ mol^−1^ (for example 20 kJ mol^−1^ for LTA).^39,40^ The physisorption of zeolites allows a lower energy penalty of ammonia release and rapid ammonia sorption kinetics.^41–43^ Therefore, the fast kinetics of gas sorption in zeolites and their excellent structural stability present a potential solution to overcome some limitations of the use of pure AEMH structures under fast NH_3_ sorption/desorption cycles.^44^

1 CaCl_2_ + 2NH_3_ ⇌ Ca(NH_3_)_2_Cl_2_

2 Ca(NH_3_)_2_Cl_2_ + 2NH_3_ ⇌ Ca(NH_3_)_4_Cl_2_

3 Ca(NH_3_)_4_Cl_2_ + 4NH_3_ ⇌ Ca(NH_3_)_8_Cl_2_

In this study, we designed zeolite–AEMH composites by impregnating CaCl_2_/SrCl_2_ into zeolite A and X granules. The zeolite retained the crystal structure after the ion-exchange and chloride-impregnation process. The resulting zeolite–AEMH composite granules were characterized by various methods to evaluate the structural stability after ammonia sorption–desorption cycles. Furthermore, the changes in the ammonia uptake capacity and ammonia sorption kinetics of the composite granules were compared, analyzed, and discussed regarding the pristine zeolites and AEMH materials.

## Experimental section

### Materials and methods

**Fig. 1:**
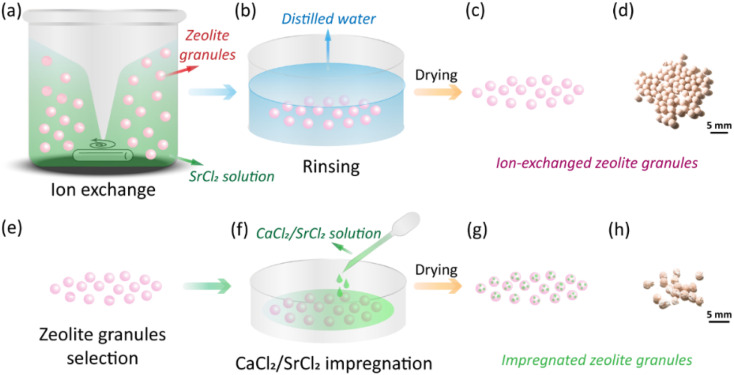
Fabrication process of zeolite–AEMH composites. (a)–(c) The ion-exchange process of CaA and NaX granules, including stirring, rinsing, and drying. (d)–(f) The AEMHs impregnation process of the selected zeolite granules, including granule selection, AEMHs impregnation, and drying.

Zeolite granules CaA and NaX (granule size 1.6–2.5 mm, Luoyang Jalong Micro-nano New Materials Co., Ltd., Henan, China), calcium chloride (anhydrous, 93% purity, Alfa Aesar) and strontium chloride (anhydrous, 99% purity, Alfa Aesar) were purchased and used as pristine materials. To reduce the formation of unexpected salts, *e.g.*, NaCl, during the impregnation process, CaA and NaX granules were first treated by ion exchange to replace the Ca^2+^ and Na^+^ cations with Sr^2+^. The impact of time on the ion-exchange process and the concentration of the SrCl_2_ solution during the ion-exchange process were investigated (Section S1, ESI†). All the ion-exchanged granules were dried at 150 °C for 1 h. As-received CaA granules were directly impregnated with CaCl_2_ solution since the formation of byproduct salts was not expected. The impregnation process was done by dripping the AEMHs solution to the granules as shown in Fig. 1(f). The loading of the AEMHs solution was from 12 wt% to 45 wt% (Section S2, ESI†). After the impregnation, the granules were dried at 150 °C for 1 h. The processing conditions of the obtained impregnated granules are listed in Table 1.

**Table 1:** The code for different impregnated granules and their processing parameters

Granule code	Composition	Ion-exchange conditions	Impregnation AEMHs loading (g^−1^ granule)
Sr_X	SrCl_2_ impregnated X	3 times with 0.40 g mL^−1^ SrCl_2_ solution	0.81 g SrCl_2_ (45 wt%)
Sr_A	SrCl_2_ impregnated A	3 times with 0.27 g mL^−1^ SrCl_2_ solution	0.27 g SrCl_2_ (21 wt%)
Ca_A_L	CaCl_2_ (low loading) impregnated A	—	0.14 g CaCl_2_ (12 wt%)
Ca_A_M	CaCl_2_ (medium loading) impregnated A	—	0.27 g CaCl_2_ (21 wt%)
Ca_A_H	CaCl_2_ (high loading) impregnated A	—	0.54 g CaCl_2_ (35 wt%)

### Structure characterization

The microstructure and elemental composition of the pristine materials and the impregnated zeolite granules were characterized by scanning electron microscopy-energy dispersive X-ray spectroscopy (SEM-EDS, JSM-IT300LV, JEOL GmbH, Germany), with a 15 nm platinum coating (Leica EM ACE 200, Germany) on the tested granules to avoid charging up from incident electrons. The crystal structure of the pristine materials and the impregnated zeolite granules was characterized by a Cu Kα radiation X-ray diffractometer (XRD, Empyrean, PANalytical, United Kingdom). All the granules were crushed and ground to a fine powder for XRD measurements. The specific surface area of the granules was obtained by N_2_ adsorption at −196 °C with the Brunauer–Emmett–Teller (BET) model using a surface area analyzer (Gemini VII 2390, Micromeritics, Norcross, USA). All measured materials were degassed at 300 °C under a dynamic vacuum overnight before the BET surface area measurements. The crushing load of the granules was measured by loading one granule (diameter 2.2 ± 0.1 mm) for a compression test using a universal machine (WDW-100, Jinan Hensgrand Instrument Co., Ltd., China), with the loading of strain rate at 1.5% s^−1^. 5 granules of each composition were measured for an average crushing load to obtain statistical reliability.

### Ammonia sorption and desorption measurement

The ammonia sorption and desorption performance were characterized by an IsoSORP® sorption analyzer (TA Instruments, United States), which consists of a magnetic suspension balance inside a chamber, an electrical heater for degassing, and a chemistry diaphragm vacuum pump to reach high vacuum. All granules were degassed at 300 °C under a high vacuum for 3 hours, following a buoyancy test with helium at 22 °C to determine the mass and volume of the tested material. Then, the ammonia sorption–desorption performance was measured with ammonia dosing from high vacuum to 1 bar (above the Sr(NH_3_)_8_Cl_2_ equilibrium pressure of 0.4 bar) for the sorption, and then back to high vacuum at the fast speed of the machine for the desorption to characterize the kinetics performance, especially in the first 10 min regime. The equilibrium of the ammonia sorption–desorption was set until the standard deviation of mass was less than 0.1 mg per 10 min. The falling AEMHs and the loose AEMHs due to volume changes on the surface of granules after the ammonia cycles were removed by a 12-mesh sieve for the second cycle of ammonia measurement. For the cyclic stability characterization, 10 cycles (counting after removing the detached AEMHs) of the ammonia test were performed after removing the falling AEMHs, with ammonia dosing from high vacuum to 3 bar for the sorption, and then back to high vacuum for the desorption.

## Results and discussion

**Fig. 2:**
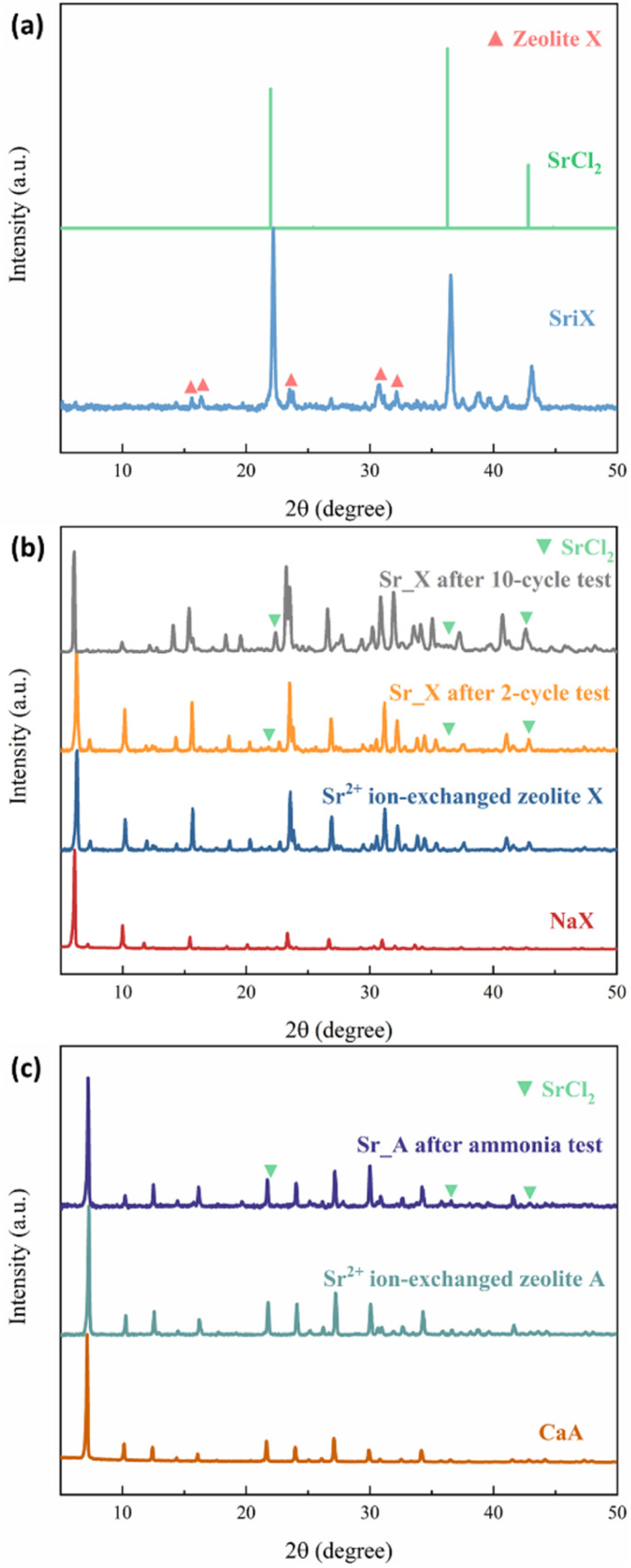
The XRD pattern of the zeolites. (a) Pristine NaX and Sr_X before ammonia test; (b) pristine NaX, Sr^2+^ ion-exchanged zeolite X, and Sr_X after 2-cycle and 10-cycle ammonia test; (c) pristine CaA, Sr^2+^ ion-exchanged zeolite A and Sr_A after ammonia test.

**Fig. 3:**
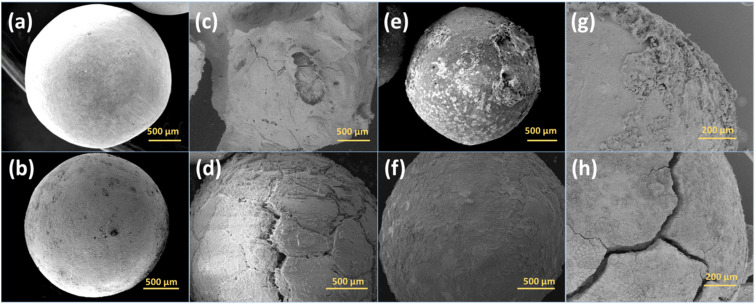
SEM images of the granules. (a) Pristine NaX; (b) pristine CaA; (c) pristine Sr_X with SrCl_2_ shell covering the zeolite X granule; (d) Sr_X after ammonia test with the SrCl_2_ shell detaching and crack formation in the granule; (e) pristine Sr_A with SrCl_2_ on the granule surface; (f) Sr_A after ammonia test with partial detachment of SrCl_2_ shell; (g) pristine Ca_A_L with CaCl_2_ on the granule surface; (h) Ca_A_L after ammonia test with partial CaCl_2_ shell detachment and crack formation in the granule.

The crystal structure of the zeolites was characterized by XRD. Sr_X contains a high SrCl_2_ loading of 45 wt%, resulting in one main peak of SrCl_2_ and weak characteristic peaks from zeolite X marked in red triangles, as shown in the X-ray diffractograms in Fig. 2(a). To verify the stability of the crystal structure of zeolites, the XRD of the impregnated granules was measured after ammonia sorption, where most of the SrCl_2_ on the surface of granules disintegrated due to volume changes and was removed by sieving. Characteristic peaks of SrCl_2_ were observed with low intensity resulting from small amounts of SrCl_2_ inside the granules and attached to the surface of the granules. After the Sr^2+^ ion exchange and SrCl_2_ impregnation process, the crystal structure of the zeolite X and A maintained the crystallinity. This is attributed to the crystal stability of the zeolite frameworks and the robust ion-exchange method.^45,46^ However, the macro-structure of the obtained granules was different before and after the ammonia sorption–desorption measurement, as shown in Fig. 3. Pristine NaX and CaA granules showed a smooth surface and a spherical morphology. Due to the high loading (45 wt%) of the SrCl_2_ in Sr_X granules, the zeolite X granules were covered by the SrCl_2_ shell (Fig. 3(c)). The amount of the SrCl_2_ shell could be controlled by the SrCl_2_ loading during the impregnation process (Section S2, ESI†). A high loading was chosen in Sr_X to achieve high ammonia uptake capacity from the AEMHs. However, due to the dramatic volume expansion (400%) of the SrCl_2_ during ammonia absorption, the thick SrCl_2_ shell fell from the zeolite X granules.^47^ Furthermore, when the SrCl_2_ inside the granules expanded, it resulted in the formation of cracks in the granule, as shown in Fig. 3(d). When the ion-exchange process was performed in high concentrated SrCl_2_ solution, cracks were formed in the CaA granules (Section S1, ESI†). Therefore, impregnation was performed at lower concentration (SrCl_2_ solution with 0.27 g mL^−1^) for the preparation of the Sr_A sample. After lowering the concentration of the impregnation solution, the SrCl_2_ loading was reduced to 21 wt% and few SrCl_2_ agglomerations were observed on the zeolite A granules as shown in Fig. 3(e). After the ammonia sorption–desorption test (Fig. 3(f)), SrCl_2_ agglomerates seem to partially detach from the surface of zeolite A granules, but crack formation in the granules was not observed, which suggests that a reduced SrCl_2_ loading in the zeolite A granules can provide better structural stability. However, in the Ca_A_L granules, with low impregnation loading (12 wt%), granule crack formation was observed, as shown in Fig. 3(h). When the CaCl_2_ loading increased as in Ca_A_M (21 wt%) and Ca_A_H (35 wt%), the degree of destruction of the granules after the ammonia test was exacerbated (Section S3, ESI†). This could be explained by the lower density of CaCl_2_ (2.15 g cm^−3^) compared with SrCl_2_ (3.05 g cm^−3^). For the same mass loading, CaCl_2_ requires more space to expand, which would result in higher stress in the granules.^15,47^

The influence of the crack formation induced by the expansion on the mechanical performance was investigated by compression test for the granules after ammonia tests. As listed in Table 2, the crushing load reduced in the impregnated granules. Particularly in Sr_X and Ca_A_L, only 43% and 17% crushing load remained after the ammonia sorption–desorption test, respectively. Sr_A retained 74% crushing load (17 N per granule) compared to the pristine CaA granules (23 N per granule), suggesting good structural stability after ammonia sorption–desorption cycles. The diametral compressive strength of the Sr_A was 4.5 MPa, which is higher than the mechanical strength reported for zeolite monoliths and granules after other treatments.^31,44^ The mechanical performance loss compared to the pristine granules could be attributed to 2 processes. First, as also reported in other studies, the repeated immersion process of the granules in the solution during the ion-exchange process might lead to a dissolution of binding points of the zeolite crystals in the granules, resulting in a drop in the compressive strength.^31^ Secondly, as shown in the SEM images in Fig. 3, the Sr_X and Ca_A_L granules showed crack formation in the granules after the ammonia sorption measurements, resulting in a lower compressive strength. The Sr_A material did not reveal such obvious crack formation and retained most of the crushing load. The Ca_A_M and Ca_A_H granules with the high CaCl_2_ impregnation loading were broken into pieces after the ammonia test, which was attributed to the large expansion during ammonia sorption–desorption cycles.

**Table 2:** The crushing load of the granules after the ammonia sorption–desorption measurement

Granules	NaX	CaA	Sr_X	Sr_A	Ca_A_L
Crushing load (N/granule)	21 ± 4	23 ± 4	9 ± 3	17 ± 5	4 ± 1

**Fig. 4:**
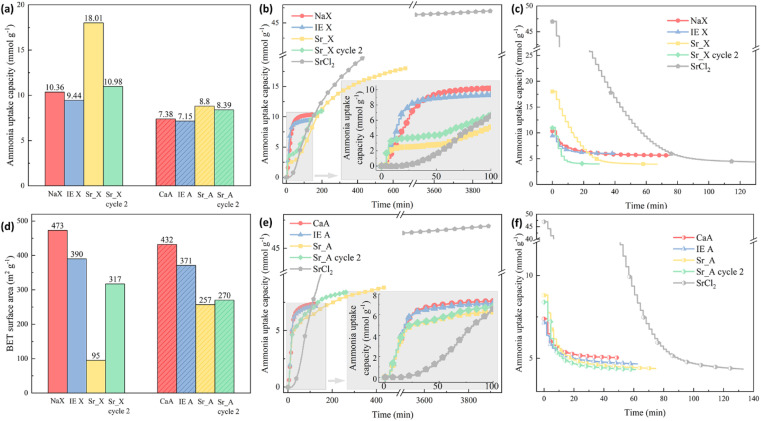
(a) The ammonia uptake capacity of the granules; (b) the ammonia sorption curves in zeolite X granule series; (c) the ammonia desorption curves in zeolite X granule series; (d) the BET surface area of the granules; (e) the ammonia sorption curves in zeolite A granule series; (f) the ammonia desorption curves in zeolite A granule series.

The ammonia sorption and desorption performance of the granules were evaluated by the ammonia uptake capacity and the kinetics as shown in Fig. 4. The experimental ammonia uptake capacity of the NaX and CaA was 10.36 mmol g^−1^ and 7.38 mmol g^−1^, respectively, which was much lower than the measured uptake of SrCl_2_ with 46.97 mmol g^−1^. After the ion-exchange process, the partially Sr^2+^ ion-exchanged zeolite X (IE X) and A (IE A) granules showed a small drop of 8% in IE X and 3% in IE A in the ammonia uptake capacity, which could be attributed to the surface area decrease after the ion-exchange process, as indicated in Fig. 4(d). On the contrary, the ammonia uptake capacity of the impregnated granules Sr_X and Sr_A increased in the first cycle despite the decrease in the surface area by 74% (18.01 mmol g^−1^) and 19% (8.80 mmol g^−1^), respectively. This can be attributed to the high ammonia capacity for samples highly impregnated with SrCl_2_.^48^ After the first ammonia sorption–desorption cycle, it was observed that the SrCl_2_ particles on the surface detached from the zeolite granules. These loose SrCl_2_ particles were removed by a 12-mesh sieve and separated from the granules. As a result of the loss of SrCl_2_ from the surface of the SrCl_2_–zeolite composite granules, the ammonia uptake capacity decreased significantly to 10.98 mmol g^−1^ and 8.39 mmol g^−1^ in Sr_X and Sr_A (green bar in Fig. 4(a)), resulting in a moderate 6% and 14% increase of the ammonia capacity compared to the pristine zeolite granules, respectively. AEMHs and zeolites were reported with an excellent cyclic performance of ammonia sorption and desorption.^33,47,49,50^ The zeolite crystal structure maintains identical after 10 cycles in Sr_X according to the XRD patterns as shown in Fig. 2, and additional detachment of AEMH material from the composite granules was not observed after the second ammonia sorption–desorption cycle, suggesting Sr_X and Sr_A possess stable structure and cyclic performance of ammonia sorption and desorption after removing the falling AEMHs. After the removal of the loose, detached SrCl_2_ particles from the composite surface by sieving, the amount of SrCl_2_ that was well attached to the Sr_X and Sr_A composite granules could be estimated from the increase in ammonia uptake compared to the ion-exchanged zeolites (without SrCl_2_ impregnation). The SrCl_2_ loading in Sr_X and Sr_A after ammonia sorption were 4 wt% and 3 wt%, respectively (for details on the calculations, see Section S4, ESI†). Fig. 4(b) and (e) reveal the kinetics of the granules in the ammonia sorption process. After the first 20 min, the pristine zeolite and both ion-exchanged zeolites, the X and the A zeolite, reached about 80% of their final, saturated ammonia uptake capacity, showing an excellent ammonia sorption kinetics. In contrast, the pure SrCl_2_ did not start to absorb ammonia before the ammonia pressure reached the equilibrium ammonia vapor pressure of 0.4 bar after 23 min.^15^ For the Sr_X and Sr_A composite granules, we observed a two-stage process of ammonia sorption. In the first 20 min (stage one), the second cycle Sr_X and Sr_A granules showed ammonia uptake capacities of 3.6 mmol g^−1^ and 4.8 mmol g^−1^, resulting in a rate of ammonia sorption at 0.18 mmol g^−1^ min^−1^ and 0.24 mmol g^−1^ min^−1^, respectively. In stage two (after 50 min), we observed ammonia uptake with a reduced sorption rate, which reflects the slower chemisorption process of ammonia sorption in SrCl_2_ compared to the physisorption process of the zeolite material in the composite. Notably, the rate of ammonia sorption in Sr_A composite granules with 0.24 mmol g^−1^ min^−1^ was 243% faster compared to SrCl_2_ with a rate of 0.07 mmol g^−1^ min^−1^ in the first 3 hours at 1 bar ammonia pressure. Such rapid kinetics in ammonia sorption in Sr_X and Sr_A composite granules can offer a quick loading of the ammonia cartridges and increase the cycle efficiency.^22^ The ammonia desorption curves of the granules were plotted in Fig. 4(c) and (g). With the pressure swing adsorption (PSA) method, no prominent difference in the desorption rate was found between granules and SrCl_2_, due to the instant high vacuum condition. However, the rapid kinetics of the physisorption of ammonia has been reported before with a temperature swing adsorption (TSA) method, where it took a relatively long time to reach the decomposition temperature for the AEMHs ammines.^34,51,52^ In our previous study, the zeolite X demonstrated 50% higher ammonia release in the first 10 min at low temperature (35 °C), suggesting the physisorbents can offer a rapid ammonia dosing in SCR at a lower temperature compared to the chemisorbents of AEMHs.^53^ The TSA measurement results show that zeolite X releases 4 times higher ammonia (0.69 mmol g^−1^) than SrCl_2_ (0.14 mmol g^−1^) in the first 10 min before reaching 60 °C (Section S5, ESI†). Therefore, combining physisorption and chemisorption in the zeolite–AEMH composites can expand the working temperature window of SCR systems. We observed that with the PSA method, the ammonia in the pristine zeolite and ion-exchanged zeolite granules demonstrated a relatively low desorption efficiency, as shown in Table 3. Less than 50% of the absorbed ammonia was desorbed in pristine NaX and CaA granules, while SrCl_2_ possessed tremendous desorption efficiency at 89%. By combining the 2 parts from zeolite and SrCl_2_, Sr_X and Sr_A granules yielded 64% and 48% desorption efficiency, respectively.

**Table 3:** Ammonia desorption capacity and the corresponding desorption percentage of the granules and SrCl_2_

Granules	NaX	CaA	Sr_X	Sr_A	SrCl_2_
Ammonia desorption capacity (mmol g^−1^)	4.67	2.34	7.02	4.07	31.03
Desorption percentage	46%	32%	64%	48%	89%

**Fig. 5:**
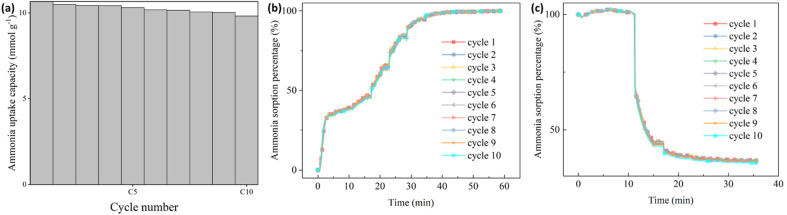
The cyclic performance of ammonia sorption and desorption in Sr_X. (a) The ammonia uptake capacity of Sr_X at 22 °C, 3 bar in 10 cycles. (b) The ammonia sorption percentage of Sr_X in the ammonia absorption for 10 cycles from high vacuum to 3 bar. (c) The ammonia sorption percentage of Sr_X in the ammonia desorption for 10 cycles from 3 bar to high vacuum.

To simulate the practical conditions for a further cyclic stability characterization, 10 cycles of ammonia sorption tests were performed at ammonia pressure at 3 bar for Sr_X. As shown in Fig. 5(a), the ammonia uptake capacity was maintained at over 92% after 10 cycles of ammonia sorption and desorption. The ammonia sorption percentage curves are identical in ammonia sorption and desorption for 10 cycles according to Fig. 5(b) and (c). The XRD patterns (Fig. 2(b)) and SEM images (Section S6, ESI†) of the Sr_X maintained stable after 10 cycles after removing the detaching SrCl_2_. All these results indicate good cyclic stability of the zeolite–AEMH composites as a long-term practical ammonia sorbent.

Based on the micro- and macrostructure, mechanical test, and the ammonia adsorption–desorption measurement, the Sr_A granule demonstrated an excellent combination between the zeolite and AEMHs, offering a solution of structural stable ammonia sorbents with rapid kinetics and elevated ammonia capacity.

## Conclusions

A simple impregnation method to combine calcium/strontium chloride and commercial zeolite A and X granules was designed. The optimized Sr_A granule demonstrated the best structural stability in terms of crystal structure and mechanical strength after ammonia sorption–desorption cycles. Moreover, Sr_A granules possessed 14% higher ammonia uptake capacity compared to the pristine CaA zeolites, and rapid ammonia kinetics with a 243% faster sorption rate than the SrCl_2_ in the first 20 min. The results confirmed two stages for the ammonia sorption in Sr_A, first physisorption followed by chemisorption. This might open a new view to solve the current challenges of the AEMHs as ammonia sorbents in SCR systems. Moreover, by adjusting the ratio of the AEMHs loading, the performance of composite granule ammonia sorbents can be tailored for various other potential applications, such as clean fuels, and hydrogen storage.

## Conflicts of interest

The authors declare no conflict of interest.

## Supplementary Material

RA-012-D2RA02981B-s001
