# Supplementary material for: Calcium/strontium chloride impregnated zeolite A and X granules as optimized ammonia sorbents
Source: RSC Adv. 2022 Dec 7;12(54):34910–7. doi: 10.1039/d2ra02981b (PMC9727750; doi:10.1039/d2ra02981b)
Supplement: RA-012-D2RA02981B-s001 [file RA-012-D2RA02981B-s001.pdf]

Electronic Supplementary Material (ESI) for RSC Advances.
This journal is © The Royal Society of Chemistry 2022

Calcium/strontium chloride impregnated zeolite A and X granules as optimized ammonia sorbents

Zhejian Cao,*[a,d] Xiaoping Cai,[b] Ana Carolina Feltrin,[a] Peizhong Feng,[b] Andreas Kaiser [c] and Farid Akhtar*[a]

[a] Division of Materials Science, Luleå University of Technology, 971 87, Luleå, Sweden. E-mail: zhejian@chalmers.se (Z.C.); farid.akhtar@ltu.se (F.A.)

[b] School of Materials Science and Physics, China University of Mining and Technology, 221116, Xuzhou, People's Republic of China

[c] Department of Energy Conversion, Technical University of Denmark, 2800 Kgs. Lyngby, Denmark

[d] Present Address: Division of Systems and Synthetic Biology, Department of Biology and Biological Engineering, Chalmers University of Technology, 41296 Gothenburg, Sweden

Electronic Supplementary Information (ESI)

S0: Safety protocol on ammonia operation and experiments

The equipment and operation involved ammonia followed lab safety protocol. The connection of the ammonia cylinder to the simultaneous IsoSORP® sorption analyzer (TA Instruments, United States) was set up with ammonia corrosion-resistant O-rings and stainless-steel pipelines. The vacuum pump for the IsoSORP® sorption analyzer was also ammonia-resistant (VA MD 1C, Vacuubrand, Germany). All the ammonia exhaust from the experiments was conducted directly to the ventilation system by pipelines and the extraction arm. The measurement setup and operation were checked and monitored by an ammonia gas detector (GAXT-A-DL, Honeywell, UK) with a sensitivity of 1 ppm.

S1: Ion-exchange process

To avoid the unexpected salt crystal produced in the impregnation process, the NaX and CaA zeolite granules were first ion-exchanged with Sr^{2+}. Based on the previously reported ion-exchange process,[1,2] the ion-exchange process was carried out by loading zeolite granules into

$SrCl_2$ solution with stirring. The magnet was put in the center bottom of the beak with a low stirring rate at 50 rpm to avoid breaking the granules. The concentration of the $SrCl_2$ solution and the bath time were investigated. The concentration of the $SrCl_2$ solution was tested at 0.14 g mL^{-1}, 0.27 g mL^{-1}, 0.40 g mL^{-1}, and 0.54 g mL^{-1}. As shown in Figure S1, at 0.54 g mL^{-1}, cracks were observed in both zeolite granules. By reducing the $SrCl_2$ concentration, the $SrCl_2$ solution was optimized at 0.40 g mL^{-1} for zeolite X and 0.27 g mL^{-1} for zeolite A.

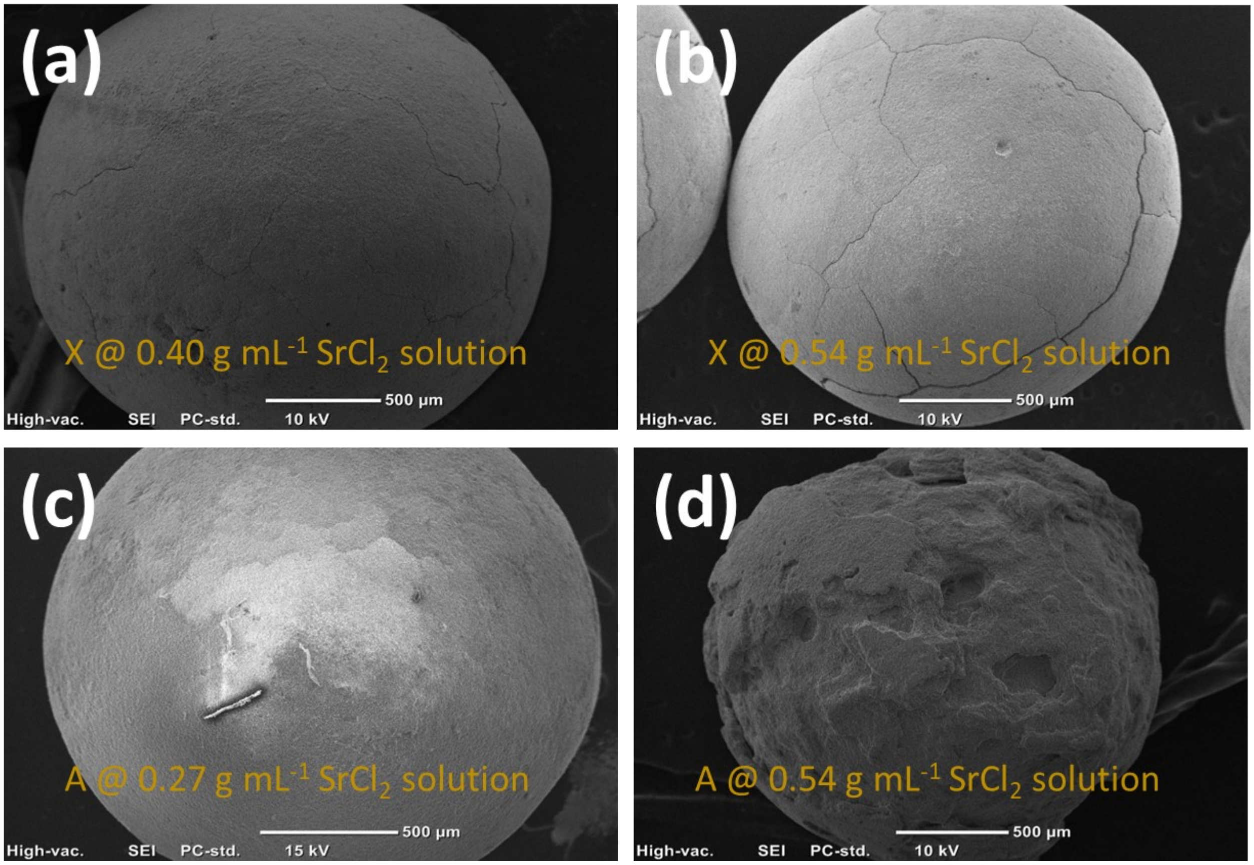

Figure S1 Ion-exchanged zeolite A and X granules with $SrCl_2$ solution at different concentration

The Sr atomic percentage in the ion-exchanged granules was measured with scanning electron microscopy–energy-dispersive X-ray spectroscopy (SEM-EDS, JSM-IT300LV, JEOL GmbH, Germany) with 5 granules for each type of zeolite to achieve statistic reliability. 30 min was regarded as one batch time for ion exchange. The repeat times of ion exchange (replace with new

$SrCl_2$ solution) were tested at 1, 2, 3, 6, and 12 cycles. From the Sr atomic percentage shown in Figure S2, we noticed that the Sr increased by 50% when comparing 1 cycle (8 at.%) to 3 cycles (12 at.%), while maintaining relatively stable at 12 cycles (14 at.%). Therefore, the ion-exchange time was repeated 3 times with 30 min each time.

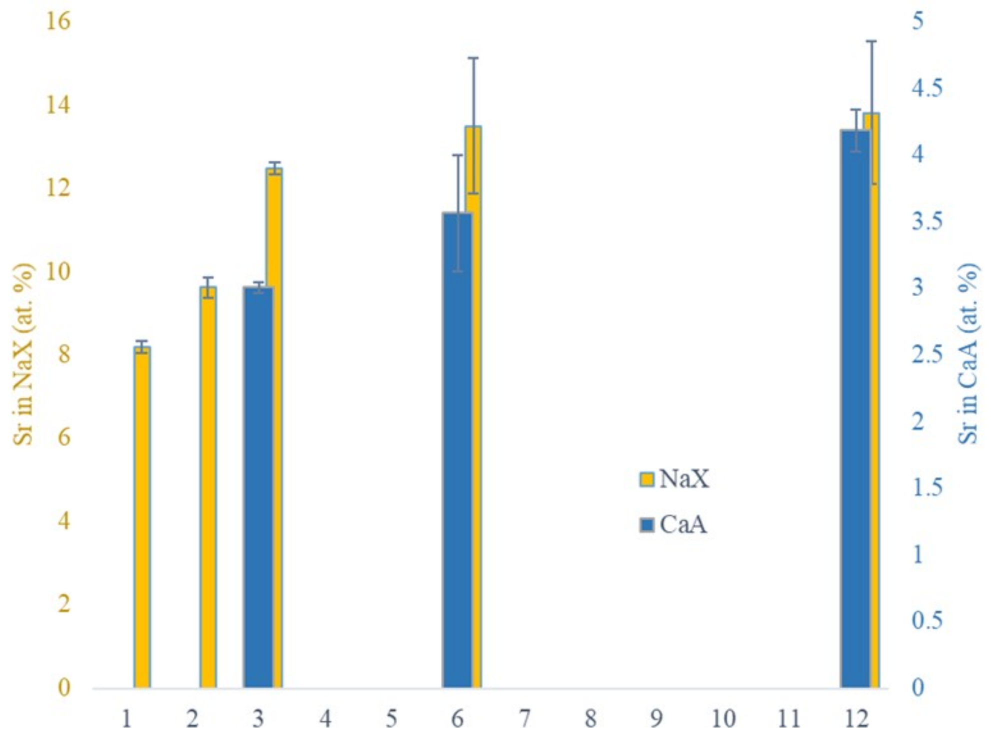

Figure S2 The Sr atomic percentage versus the number of cycles of the ion-exchange process

S2: Impregnation process

The AEMHs loading in the final composite is calculated based on the mass fraction according to Equation (S1), where ω_{AEMHs}, the mass fraction of AEMHs; m_{AEMHs}, the mass of the AEMHs; $m_{zeolite}$, the mass of zeolite.

$$\omega_{AEMHs} = \frac{m_{AEMHs}}{m_{AEMHs} + m_{zeolite}} \times 100\% \quad \text{(S1)}$$

The $SrCl_2$ loading can be adjusted by dripping different amounts of $SrCl_2$ solution into the granules. For the zeolite granule X, the 0.54 g ml^{-1} $SrCl_2$ solution was used, and 0.27 g ml^{-1} $SrCl_2$ solution was used for zeolite A as verified in section S1. As shown in Figure S3, by dripping 1.5 mL 0.54 g ml^{-1} $SrCl_2$ solution into 1 g ion-exchanged zeolite X, we obtained 0.81 g $SrCl_2$ loading (45 wt%) in Sr_X. For Sr_A, 1 mL 0.27 g ml^{-1} $SrCl_2$ solution into 1 g ion-exchanged zeolite A.

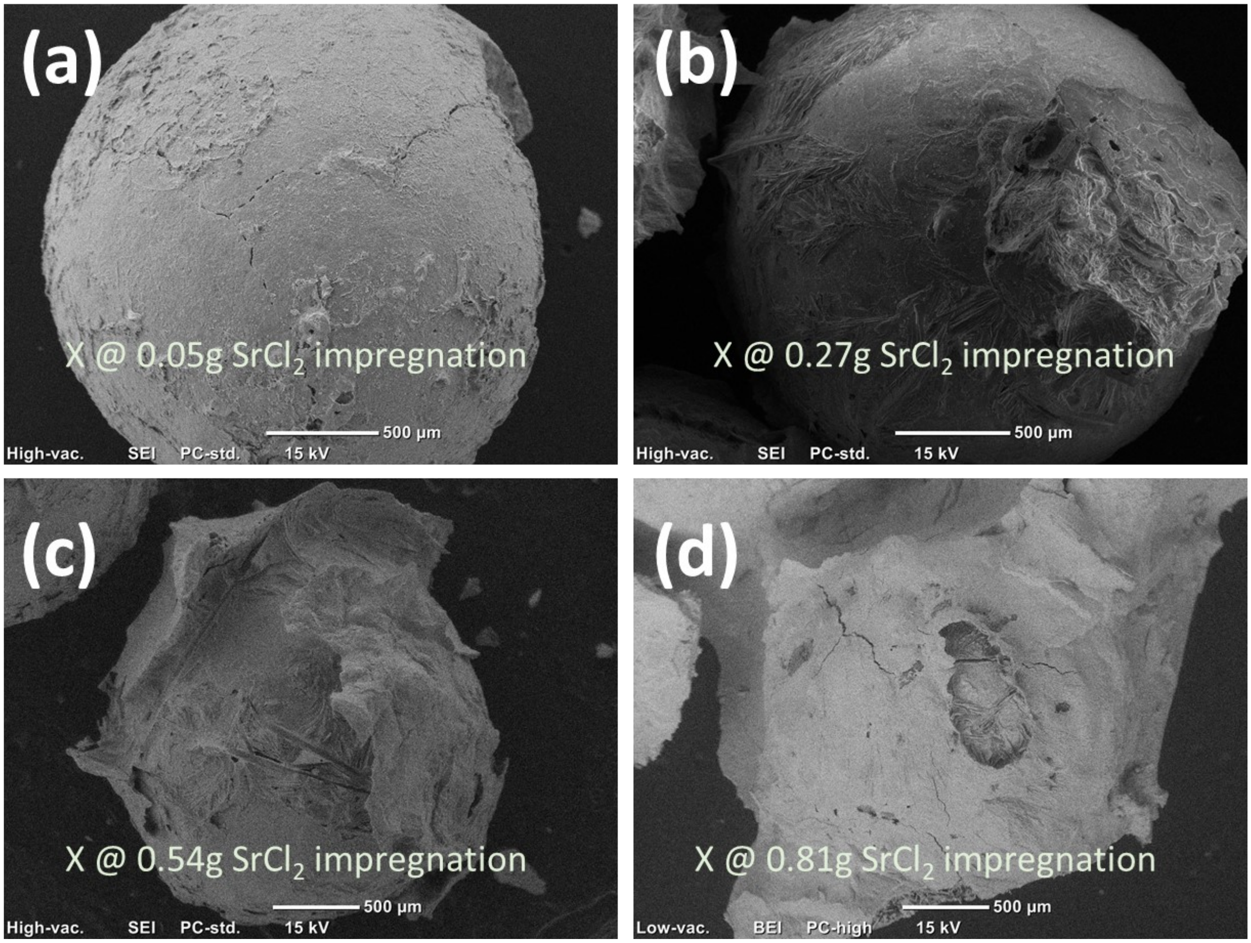

Figure S3 The impregnated zeolite X granules with different $SrCl_2$ loading

S3: Destruction of the Ca_A_M and Ca_A_H granules

Due to the expansion of $CaCl_2$ during ammonia sorption, Ca_A_M (21 wt% $CaCl_2$) and Ca_A_H (35 wt% $CaCl_2$) demonstrated severe structure disintegration, as shown in Figure S4

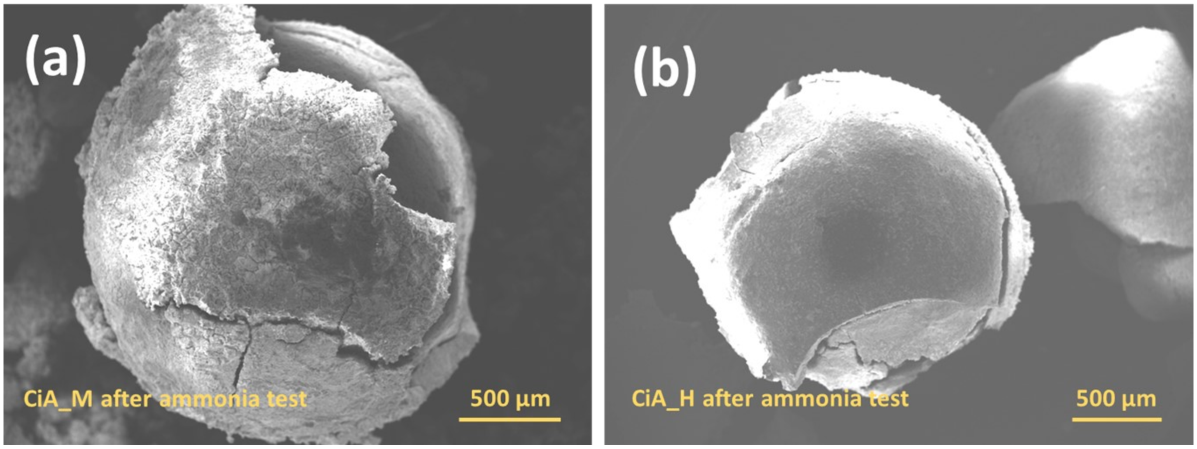

Figure S4 The SEM images of the Ca_A_M and Ca_A_H granules after ammonia adsorption-desorption measurement

S4: Estimation of the $SrCl_2$ loading after ammonia sorption and sieving

The ammonia uptake capacity of the impregnated granules has been contributed from two parts, the $SrCl_2$, and the zeolite. If we assume the ammonia uptake capacity of the impregnated granules (M_{final}) is composed proportional to the $SrCl_2$ (M_{SrCl2}) and ammonia uptake capacity of the zeolite ($M_{zeolite}$) and according to their corresponding mass fraction (ω_{SrCl2} and $\omega_{zeolite}$, where $\omega_{SrCl2} + \omega_{zeolite} = 1$) in equation (S2), we can calculate the actual $SrCl_2$ loading (ω_{SrCl2}) based on the measured M_{SrCl2}, 46.97 mmol g^{-1}, $M_{zeolite}$, 9.44 mmol g^{-1} and 7.15 mmol g^{-1} for ion-exchanged X and A, respectively, yielding 4 wt% and 3 wt% SrCl2 loading in Sr_X and Sr_A after removing the falling and loose salts on the granule surface.

$$M_{final} = M_{SrCl2} * \omega_{SrCl2} + M_{zeolite} * \omega_{zeolite} \qquad \text{(S2)}$$

S5: Isobaric curves of ammonia desorption by temperature swing adsorption (TSA) method

To mimic the current practical ammonia desorption process, the ammonia desorption performance of the materials was characterized by the temperature swing adsorption (TSA) method.[3] The saturated ammonia sorbents were maintained at a 3 bar ammonia atmosphere, and the temperature of the reaction chamber was increased to 120 °C by the electrical heater to achieve the fast ramp speed in the machine (~3 °C min^{-1}). As shown in Figure S5(a), in the first 10 min, zeolite X released 7.1% ammonia (0.69 mmol g^{-1}), which is around 4 times higher than 0.3% in $SrCl_2$ (0.14 mmol g^{-1}). The Sr_X after 2-cycle was observed to have faster kinetics in the ammonia sorption kinetics before the temperature reaches the $Sr(NH_3)_8Cl_2$ decomposition temperature of 60 °C as shown in the blue regime in Figure S5(b). After the temperature surpasses 60 °C, each $Sr(NH_3)_8Cl_2$ releases 7 ammonia molecules (87.5%), giving abundant ammonia dosing.

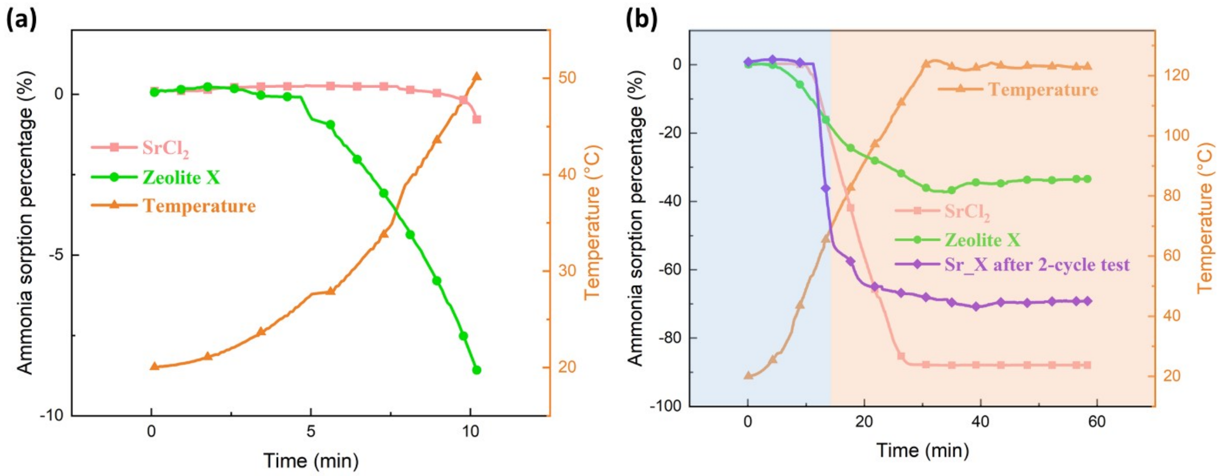

Figure S5 The isobaric ammonia sorption percentage curves in the ammonia desorption at 3 bar, with temperature increasing from room temperature (~20 °C) to 120 °C. (a) ammonia sorption percentage of $SrCl_2$ and zeolite X in the first 10 min before the temperature reaches 60 °C, (b) ammonia sorption percentage of $SrCl_2$, zeolite X, and Sr_X after 2-cycle test, the blue region below 60 °C, the orange region above 60°C.

S6: Structural stability of Sr_X after 10 cycles of ammonia sorption and desorption

After removing the detached $SrCl_2$ from the first ammonia test, some cracks were found in the zeolite granule. By repeating 10 more cycles after that, no obvious extra cracks were found, as shown in Figure S6, suggesting good cyclic structural stability.

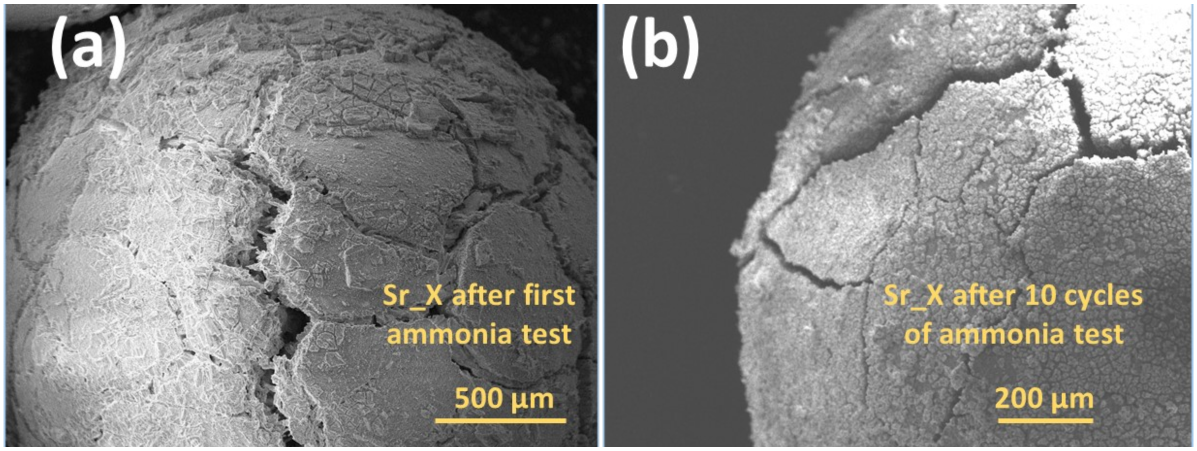

Figure S6 The SEM images of the Sr_X granules after (a) the first ammonia test and (b) after 10-cycle of the ammonia test

References

1 O. Cheung, Z. Bacsik, Q. Liu, A. Mace, and N. Hedin, *Applied Energy*, 2013, **112**, 1326–1336.
2 K. Narang, K. Fodor, A. Kaiser, and F. Akhtar, *RSC Adv.*, 2018, **8**, 37277–37285.
3 S. Wu, T. X. Li and R. Z. Wang, *Energy*, 2018, **161**, 955–962.
